# From cheek to ureter: initial experience and learning curve of single-port robotic buccal mucosa graft ureteroplasty for complex strictures

**DOI:** 10.1007/s11701-026-03488-1

**Published:** 2026-05-21

**Authors:** Jin-chun Qi, Hao-xuan Yang, Dong-bin Wang, Sheng Tang, Run-long Wang, Lorenzo Santodirocco, Marwan Alkassis, Ruben Sauer Calvo, Wen-yong Xue, Ya-Xuan Wang, Simone Crivellaro

**Affiliations:** 1 Department of Urology, The Second Hospital of Hebei Medical University, No. 215 Heping West Road, Shijiazhuang, Hebei China; 2 Department of Urology, University of Illinois at Chicago, Chicago, IL USA; 3 Department of Maternal-Infant and Urological Sciences, Sapienza University of Rome, Umberto I Hospital, Rome, Italy

**Keywords:** Single-port robot, Buccal mucosa graft, Ureteral stricture, Ureteroplasty, Learning curve

## Abstract

To evaluate the initial experience of single-port robot-assisted ureteral reconstruction using a buccal mucosa graft and assess the preliminary learning curve associated with this technique. A retrospective analysis was conducted on 20 consecutive patients who underwent single-port robot-assisted ureteroplasty with buccal mucosa grafts between January 2020 and January 2026. Perioperative parameters, complications, and follow-up outcomes were recorded. Restenosis-free survival was estimated using the Kaplan–Meier method, and the learning curve for operative time was analyzed using the cumulative sum (CUSUM) method. All procedures were completed without conversion. The median follow-up period was 4.5 months (range, 1–12). The overall success rate was 90% (18/20), with two patients developing restenosis at 4 and 7 months postoperatively, requiring reintervention. The 30-day complication rate was 20% (4/20), including one Clavien grade II and three grade IIIa complications. Long-term complications occurred in two patients (10%). CUSUM analysis demonstrated a notable decline in operative time after approximately 10 cases, with stabilization after 17 cases. Single-port robot-assisted ureteroplasty with buccal mucosa grafting appears to be a safe and effective minimally invasive option for complex ureteral strictures, with favorable initial experience and an acceptable learning curve. Larger prospective studies with extended follow-up are required to confirm the long-term efficacy.

## Introduction

 Ureteral strictures remain a challenging clinical entity, commonly resulting from iatrogenic injury, radiation therapy, congenital anomalies, or idiopathic causes [1]. For short ureteral strictures, treatments such as balloon dilation, ureteroscopic stricture incision, and resection with end-to-end anastomosis are often employed [2, 3]. However, in cases of recurrent, multifocal, or long-segment (> 2 cm) strictures, primary anastomosis may be precluded by excessive tension. In such scenarios, reconstruction using intestinal segments, bladder flaps, or even auto-transplantation may be considered. However, these procedures are associated with significant morbidity [4].

In 1999, Naude first reported the use of buccal mucosa for ureteral repair, demonstrating promising outcomes [5]. The buccal mucosa shares several characteristics with the urothelium, including a thick, non-keratinized epithelium and well-vascularized lamina propria. It is easy to harvest, resistant to infection, and associated with low donor site morbidity, making it an attractive graft material for ureteral reconstruction [6–8].

Robotic-assisted laparoscopy has enabled more precise ureteral reconstruction with advances in minimally invasive surgery. The introduction of single-port robotic platforms represents further evolution, offering reduced surgical trauma and improved cosmetic results. Data on initial experience and learning curves remain scarce, particularly for single-port systems, although early reports have confirmed the feasibility of robotic ureteroplasty with buccal mucosa grafts [9–13]. This study aimed to describe the surgical technique, evaluate perioperative and early functional outcomes, and characterize the learning curve during the adoption phase of this approach.

## Materials and methods

### Study population

This single-center retrospective study included 20 consecutive patients who underwent single-port robot-assisted ureteroplasty with buccal mucosa grafts between January 2020 and January 2026 at our institution. All procedures were performed by a single surgeon with over 10 years of experience in minimally invasive urological surgeries. The final follow-up was conducted in February 2026.

The collected data included patient demographics (age, sex, BMI, and side of surgery), stricture characteristics (etiology, location, and length), perioperative details (operative time, estimated blood loss, hospital stay, graft length, and double-J stent duration), and complications (classified according to the Clavien–Dindo classification). Follow-up assessments included symptom evaluation, renal function tests, ultrasonography, CT urography (CTU), and diuretic renography.

Surgical success was defined as the absence of symptoms, no evidence of restenosis on imaging (diuretic renography T½ < 20 min or patent ureter on CTU), and no need for reintervention. Failure was defined as symptomatic or radiological restenosis requiring balloon dilation, stent replacement or revision surgery. This study protocol was approved by the Institutional Review Board (IRB) of the University of Illinois at Chicago (IRB: 2021-0012) and was updated annually.

The inclusion criteria were as follows: (1) age ≥ 18 years; (2) ureteral stricture length ≥ 2 cm; (3) no prior treatment for the stricture; and (4) surgery performed by the designated surgeon using the da Vinci SP platform. For strictures of 2 cm or longer, primary resection and end-to-end anastomosis often carry a high risk of tension-induced failure or restenosis, making it unsuitable; therefore, a graft-based reconstruction such as buccal mucosa ureteroplasty is preferred.

The exclusion criteria were as follows:: (1)follow-up duration of less than one month; (2)bilateral ureteropelvic junction obstruction (UPJO) or a history of ipsilateral renal/ureteral surgery; (3)presence of other urological diseases requiring concurrent surgical intervention; (4)severe systemic diseases that could potentially affect the evaluation of surgical outcomes; (5)intraoperative conversion to open or multiport laparoscopic surgery; and (6) incomplete perioperative clinical data that precluded the assessment of primary outcome measures.

### Surgical technique (Fig. 1)

All procedures were performed using the da Vinci SP^®^ robotic system. The standard instrument configuration included a 12-mm endoscope channel, two articulated monopolar curved scissors, one Cadiere forceps, and one fenestrated bipolar forceps. Patients were positioned in the flank position, and single-port transperitoneal access was established. The ureter was mobilized, and the stricture was identified and measured. A buccal mucosa graft of appropriate length was harvested from the inner cheek or lower lip, defatted, and trimmed into a patch or tubular configuration. The strictured segment was incised longitudinally, and the graft was anastomosed using the onlay technique. Complete transection with tubularized graft reconstruction was performed in select cases. A double-J stent was placed and typically removed 4–8 weeks postoperatively, depending on the recovery.

**Fig. 1:**
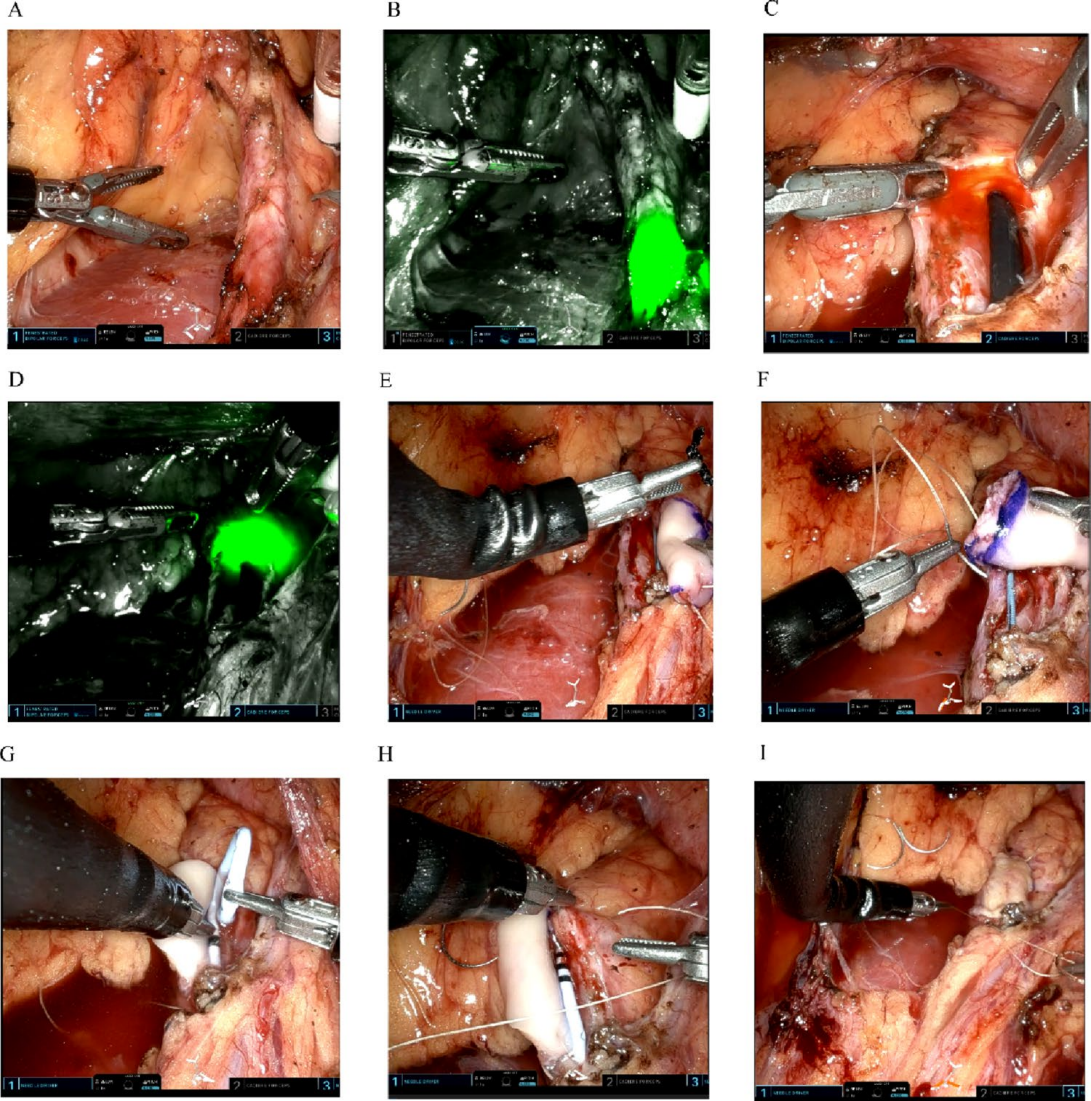
Right proximal ureteral stricture repair with buccal mucosa graft single-port robot. (**A**) Right ureteral stricture identified. (**B**) Firefly fluorescence imaging confirms ureteroscope position at stricture. **C, D** Incision of Ureteral Stricture to Ureteroscope Passage, Confirmed by Firefly Fluorescence Imaging. **E-I** Continuous Onlay of Buccal Mucosa Graft for Ureteral Stricture Repair with Stent Placement

### Statistical analysis

The patients were numbered sequentially according to operative date. The cumulative sum (CUSUM) method was used to analyze the learning curve for operative time, with the target set at the median operative duration. Continuous variables are expressed as mean ± standard deviation or median (range), as appropriate. Categorical variables are presented as frequencies and percentages. Restenosis-free survival was estimated using the Kaplan–Meier method. All analyses were conducted using SPSS version 27.0, with statistical significance set at *p* < 0.05.

## Results

### Baseline characteristics and surgical outcomes

The cohort included 13 men (65%) and 7 women (35%), with a mean age of 47.6 ± 16.2 years and a mean BMI of 28.2 ± 7.7 kg/m². The most common procedure was single-port robot-assisted laparoscopic ureteroplasty with buccal mucosa graft (*ureteroplasty w/BMG*) (9 cases, 45%), followed by single-port robot-assisted laparoscopic pyeloplasty with buccal mucosa graft (*pyeloplasty w/BMG*) (7 cases, 35%). Additionally, there were three cases of single-port robot-assisted laparoscopic bladder neck repair with buccal mucosa graft (these patients were included because the surgical principle of onlay buccal mucosa grafting for luminal reconstruction is identical to that of ureteroplasty, and their inclusion allowed us to analyze the learning curve across anatomically similar procedures) and one case of single-port robot-assisted laparoscopic pyeloplasty combined with ureteroureterostomy using buccal mucosa graft (Table 1).

**Table 1:** Comparison of Patient Baseline Characteristics and Surgical Outcomes

Parameter	Value
Age (years, x̄ ± s)	47.6 ± 16.2
Gender (Male/Female, n)	13/7
BMI (kg/m², x̄ ± s)	28.2 ± 7.7
Type of surgery (n, %)	
*Pyeloplasty w/BMG*	7(35)
*Ureteroplasty w/BMG*	9(45)
*Bladder neck repair w/BMG*	3(15)
*Pyeloplasty + Ureteroplasty w/BMG*	1(5)
Stricture Etiology (n, %)	
*Primary*	4 (20)
*Iatrogenic injury*	12 (60)
*Other*	4 (20)
Stricture Side (Left/Right, n)	9/11
Stricture Location (n, %)	
*Proximal*	5 (46)
*Mid*	2 (18)
*Distal*	4 (36)
Stricture Length (cm, x̄ ± s)	2.6 ± 0.7
Surgical Details	
*Prior Abdominal/Pelvic Surgery History (n*,* %)*	16 (80)
*Operative Time (min*,* x̄ ± s)*	187 ± 61
*Estimated Blood Loss (ml*,* x̄ ± s)*	29.8 ± 15.9
*Hospital Stay (hours*,* x̄ ± s)*	20.6 ± 17.7
Foley Indwelling Time (days, Median [Range])	7(2,28)
Double-J Stent Indwelling Time (weeks, Median [Range])	8 (4,17)

Iatrogenic injury was the most common etiology (12cases, 60%), including five post-radiation and seven post-surgical strictures. Primary strictures accounted for four cases (20%), and other causes accounted for four cases (20%). The stricture was left-sided in 9 (45%) and right-sided in 11 (55%) patients. Stricture locations included proximal (*n* = 5, 46%), mid (*n* = 2, 18%), and distal (*n* = 4, 36%). The mean stricture length was 2.6 ± 0.7 cm.

All procedures were completed without conversion to open surgeries. The mean operative time was 187 ± 61 min, mean estimated blood loss was 29.8 ± 15.9 mL, and mean hospital stay was 20.6 ± 17.7 h. A Foley catheter was placed in 16 patients, with a median indwelling time of 7 days (range, 2–28). Among the 13 patients who had their double-J stents removed, the median indwelling time was 8 weeks (range, 4–17). Two patients retained their stents at the time of analysis, and two had not yet been removed.

### Complications

Four patients (20%) experienced complications within 30 days: one Clavien grade II (acute kidney injury) and three grade IIIa (retroperitoneal fluid collections managed with percutaneous drainage). For patients with persistent drainage, routine laboratory evaluations included body fluid cell count, body fluid culture, and fluid creatinine level, along with simultaneous serum creatinine measurement to differentiate urinoma from other fluid collections, assess cellular content, and rule out infection. In all three cases, the fluid creatinine level exceeded twice the serum creatinine level, confirming the diagnosis of a urinoma. For example, in one representative patient, the fluid creatinine level was 6.75 mg/dL, while the serum creatinine level was 0.75 mg/dL (ratio 9:1), which is highly suggestive of urine leakage/urinoma. No secondary infections were observed. Long-term complications occurred in two patients (10%): one required balloon dilation for restenosis at 4 months, and the other underwent nephrectomy for refractory restenosis at 7 months. No Clavien grade IV or V complications were observed.（Table 2）

### Restenosis-free survival

**Fig. 2:**
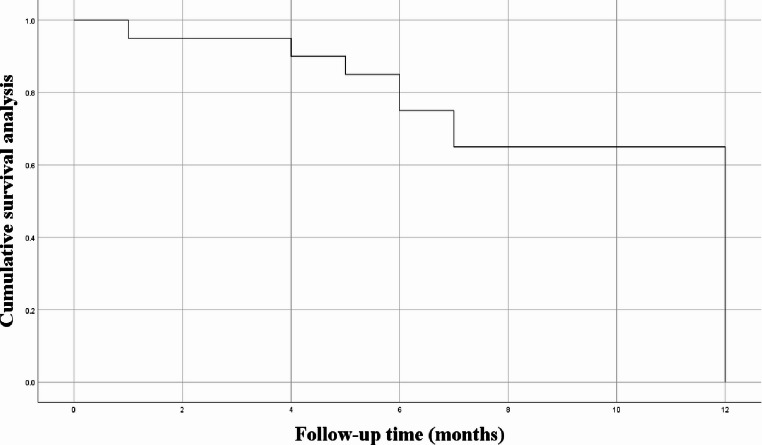
Restenosis-free survival curve

The median follow-up period was 4.5 months (range, 1–12 months). The overall success rate was 90% (18/20). Kaplan–Meier analysis showed a 3-month restenosis-free survival rate of 95% and a 1-year rate of 90% (Fig. 2).

### Learning curve

**Fig. 3:**
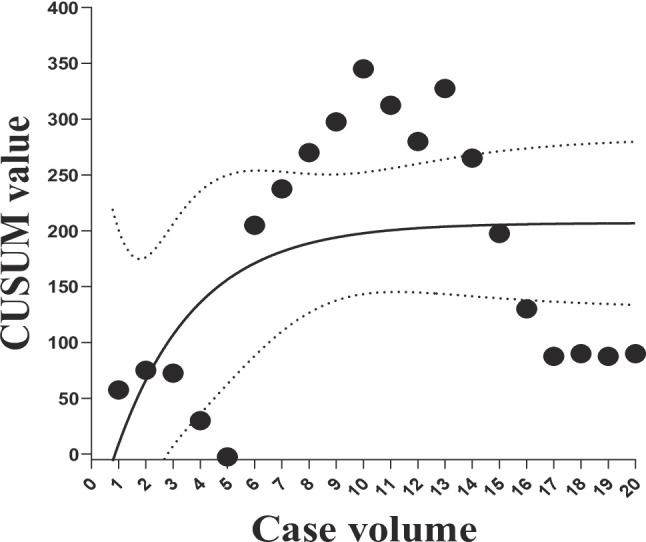
CUSUM learning curve for operative duration

The CUSUM analysis of operative time is shown in Fig. 3. Using a target value of 182.5 min (the median operative time), the CUSUM curve declined after the first three cases but rose again after a difficult case (case 6). The sixth case was the surgeon’s first attempt at single-port robot-assisted laparoscopic bladder neck repair with a lingual mucosal graft. It peaked at case 10 and then gradually declined, stabilizing after approximately 17 cases, indicating that the learning curve had plateaued after 17 procedures. The increase at the learning curve at the 13th case was attributed to the fact that this case involved a single-port robot-assisted laparoscopic pyeloplasty combined with ureteroureterostomy using a buccal mucosa graft, which required a longer operative time than either procedure alone (Fig. 3).

## Discussion

The treatment of long-segment ureteral strictures remains challenging. Common treatment modalities include ileal ureter replacement and autologous kidney transplantation, which are complex procedures associated with high surgical risks and significant complications; hence, neither is entirely satisfactory. Recent population-based trend analyses have shown that minimally invasive reconstructive techniques are increasingly being adopted for ureteral repair [14]. The suitability of the buccal mucosa as a substitute material for the ureter stems from its anatomical characteristics: a thick, non-keratinized epithelium and highly vascularized lamina propria. These features contribute to its ease of survival, lack of rejection, absence of metabolic disturbances, ease of harvest, rapid donor site healing, and low rate of harvest site complications, making it widely used for urinary tract reconstruction [15–18]. Ureteroplasty using buccal mucosa grafts has gradually become minimally invasive due to advances in laparoscopic techniques. These approaches have made ureteral repair and reconstruction safer and less traumatic, with various countries reporting laparoscopic or robot-assisted laparoscopic procedures [19–21]. Recently, the emergence of single-port laparoscopic robots has further advanced ureteral reconstruction techniques to a new peak of minimally invasive procedures.

Previous independent case reports on single-port robotic-assisted lingual mucosal graft ureteroplasty for treating ureteral strictures have preliminarily demonstrated the practicality and feasibility of this procedure [22]. However, further research is required to validate its safety and learnability. The present study provides early evidence from an initial experience, demonstrating that this technique is feasible and can be safely implemented in a selected cohort of patients. With an overall success rate of 90%, a 1-year recurrence-free survival rate of 90%, a median operative time of 182.5 min, and a postoperative complication rate of 20%, all of which are similar to those in our previous study [23].

**Table 2:** Postoperative complications

Complication	Number of Cases (%)	Clavien-Dindo Grade
30-Day Complications	4 (20)	
*Acute Kidney Injury*	1 (5)	II
*Retroperitoneal Fluid Collection*	3 (15)	IIIa
Long-term Complications	2 (10)	
*Restenosis treated with balloon dilation*	1 (5)	IIIa
*Restenosis requiring nephrectomy*	1 (5)	IIIb

Six patients experienced complications. Four patients underwent surgery within 30 days (20%), predominantly Clavien grade IIIa or lower, and no severe complications were observed. These included three cases of retroperitoneal fluid collection and one case of AKI, all of which were managed promptly, indicating the safety of the technique. Two patients experienced long-term restenosis. One patient with a bladder neck stricture had previously undergone Robot-Assisted Radical Prostatectomy (RARP) and had a permanent cystostomy. After treatment of the bladder neck stricture with buccal mucosa graft, symptoms recurred at 4 months postoperatively, which improved after balloon dilation. Another patient had a history of bilateral pyeloplasty. The patient presented with a 4 cm right ureteral stricture and was treated with buccal mucosa graft ureteroplasty. However, renal function did not improve postoperatively, and nephrectomy was performed after 7 months. Therefore, postoperative restenosis may be associated with factors such as excessive stricture length, poor vascular supply, and graft ischemia. We recommend strict adherence to the indications and close postoperative follow-up, especially in patients with long-segment strictures (> 3 cm) or those with a history of radiation therapy.

Learning curve analysis showed that operative time began to decrease after approximately three cases. However, in the 6th case, intraoperative difficulties significantly prolonged the operative time. Consequently, the CUSUM value increased in subsequent cases, peaking at the 10th case, then declining and stabilizing around the 17th case. This suggests that for surgeons experienced in laparoscopy and robotic surgery, becoming proficient with the single-port robot and performing single-port robotic procedures is not particularly difficult, and an ideal operative time can be achieved relatively quickly. In this study, the sixth case was the surgeon’s first attempt at single-port robot-assisted laparoscopic bladder neck repair using a lingual mucosal graft. The surgeon encountered intraoperative difficulties, resulting in a significantly prolonged operative time, and the operative times for the subsequent few cases were also relatively extended. Encountering difficulties during surgery can significantly prolong the operative time in subsequent cases; however, through continued learning, the operative time gradually decreases and becomes stable. Longer operative times in the initial cases may be related to unfamiliarity with the intraoperative adjustments and graft fixation techniques. As experience accumulates, anastomotic efficiency improves, and operative time decreases. The final CUSUM curve did not reach zero, indicating the potential for further improvement beyond the current series. The mean operative time was 187 min, and the median was 182.5 min, suggesting that the typical duration for single-port robot-assisted laparoscopic ureteroplasty with buccal mucosa graft is approximately 180–190 min. Once a surgeon consistently achieves operative times within or below this range, they can be considered to have mastered the procedure.

In single-port robot-assisted laparoscopic buccal mucosa graft ureteroplasty, fluorescence imaging (Firefly) provides essential visual support during critical steps, greatly improving both precision and safety. This technology employs real-time fluorescence to clearly outline the extent of ureteral strictures, helping surgeons accurately identify lesion boundaries amid inflamed and fibrotic tissue. This ensures that the incision and reconstruction precisely cover the diseased segment while avoiding excessive resections or residual strictures. Additionally, Firefly allows the evaluation of blood perfusion at the ureteral margins and surrounding tissues before graft placement, guiding the surgeon to perform anastomosis in well-vascularized areas and reducing the risk of poor healing or restenosis caused by ischemia. In the limited space of single-port robotic surgery, this technology converts critical anatomical information into intuitive imaging, improving the surgeon’s ability to navigate complex anatomy and enabling more precise positioning and suturing of the buccal mucosa graft. Ultimately, this enhances the reconstructive quality and leads to better surgical outcomes.

This study had a single-center, retrospective design with a small sample size, which may have led to selection bias. The surgeon involved was an experienced urologist proficient in laparoscopic and robotic techniques; the learning curve and number of cases required to master this procedure may not be directly applicable to beginners with limited or no experience in laparoscopic or robotic surgery. Future large-sample, multicenter, prospective studies involving surgeons with varying levels of experience are needed to further validate the efficacy of this technique and to explore the application of tissue engineering materials in ureteral reconstruction. Our findings may serve as a foundation for subsequent research mentioned above.

## Conclusion

Single-port robot-assisted ureteroplasty with buccal mucosa grafts demonstrated a favorable initial experience in our study, with an acceptable learning curve trend. This technique can be considered a minimally invasive treatment option for complex ureteral stricture. However, studies with larger sample sizes and longer follow-up periods are required to confirm the long-term outcomes.

## Data Availability

All data supporting the findings of this study are available within the paper and its Supplementary Information.

## Abbreviations

AKI: Acute Kidney Injury
BMI: Body Mass Index
BMG: Buccal Mucosa Graft
CTU: Computed Tomography Urography
CUSUM: Cumulative Sum
IRB: Institutional Review Board
RARP: Robot-Assisted Radical Prostatectomy
SP: Single-Port
UPJO: Ureteropelvic Junction Obstruction
